# Experience of comprehensive sequential therapy for salivary duct carcinoma with HER2 gene amplification: a case report

**DOI:** 10.1016/j.bjorl.2026.101765

**Published:** 2026-02-06

**Authors:** Yu Zhang, Chenke Wei, Wei Shang, Zongxuan He

**Affiliations:** a The Affiliated Hospital of Qingdao University, Department of Oral and Maxillofacial Surgery, Shandong Province, China; b Qingdao University, School of Stomatology, Shandong Province, China

## Introduction

Salivary Duct Carcinoma (SDC) originates from the ductal epithelium of the salivary glands and represents approximately 1%–3% of all malignant tumours affecting these glands. Reports suggest that 65.4% of patients with SDC experience postoperative recurrence or distant metastasis, with approximately 42% succumbing within 5-years,^1^ therefore impacting prognosis. Currently, most literature indicates that SDC predominantly occurs in the parotid gland, with submandibular gland involvement being relatively rare. The preferred treatment options for advanced-stage SDC involve complete tumour resection followed by neck lymph node dissection coupled with adjuvant radiotherapy. Some studies reveal prevalent mutations in TP53 (56%), PIK3CA (33%), and HRAS (33%) genes in salivary duct carcinoma.^2^ When administered judiciously, targeted therapy demonstrates favourable efficacy. In this case report, we report a successful case of advanced Human Epidermal Growth Factor Receptor 2 (HER2)-positive submandibular gland ductal carcinoma treated with trastuzumab in combination with a paclitaxel and cisplatin (TP) regimen.

## Case report

**Fig. 1:**
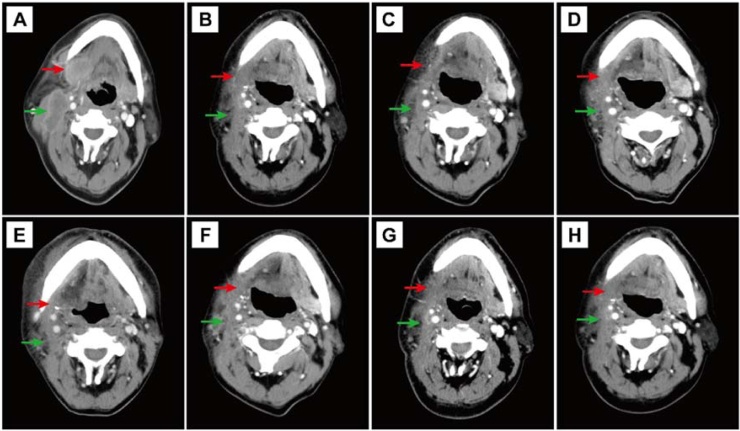
Contrast-enhanced CT scans demonstrating the tumour (20 × 25 × 32 mm) located in the right submandibular gland area (red arrow) and multiple enlarged lymph nodes (green arrow) in the right neck (A). Follow-up contrast-enhanced CT scans at 1-month (B), 6-months (C), 12-months (D), 15-months (E), 21-months (F), 27-months (G), and 36-months (H).

**Fig. 2:**
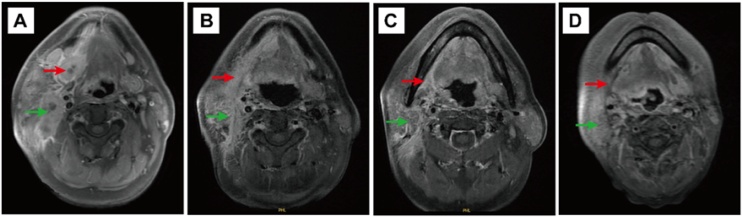
Magnetic Resonance Imaging (MRI) revealing the tumour (red arrow) in the right submandibular gland with suspicious metastatic lymph nodes (green arrow) (A). Follow-up MRI scans at 12-months (B), 24-months (C), and 36-months (D).

The Department of Oral and Maxillofacial Surgery received a 53-year-old male patient complaining of right submaxillary swelling and pain persisting for one week. Upon physical examination, a firm mass was palpated in the right submaxillary region alongside notable lymph nodes in the ipsilateral neck area. Contrast-enhanced Computed Tomography (CT) and Magnetic Resonance Imaging (MRI) of the neck confirmed a malignant tumour in the right submandibular gland with lymph node metastasis. Furthermore, central necrosis was identified in one of the lymph nodes (Fig. 1A and Fig. 2A).

**Fig. 3:**
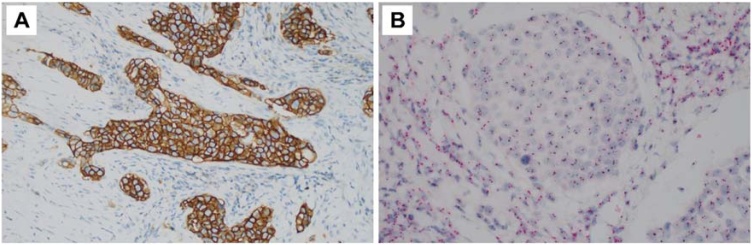
Immunohistochemical staining for HER2 showing positive tumour cells (A, ×200). Dual Colour in Situ Hybridization (DISH) of HER2/CEP17 indicating HER2 gene amplification in black signals and the centromeric region of Chromosome 17 (CEP17) in red signals (B, ×200).

The patient underwent ultrasound-guided core needle biopsy of the submandibular gland mass. An 18 G needle was used, and the obtained specimen, with a length of 1.5 cm, was immediately sent for pathological examination. The diagnosis of Salivary Duct Carcinoma (SDC) was confirmed by Hematoxylin-Eosin (HE) staining. In accordance with the National Comprehensive Cancer Network Clinical Practice Guidelines in Oncology, surgical planning was firstly undertaken. The patient underwent excision of the right-sided submandibular gland and a neck dissection encompassing Levels I‒V. Postoperative pathological analysis confirmed the diagnosis of SDC, classified as pT4bN3bM0 according to the American Joint Committee on Cancer TNM staging system. Immunohistochemistry (IHC) in September 2021 demonstrated Androgen Receptor (AR) positivity (10%) and HER2 overexpression (3+) (Fig. 3A). Further molecular genetic analysis utilizing Dual In Situ Hybridization (DISH) and Next-Generation Sequencing (NGS) unveiled HER2 amplification (Fig. 3B).

A multidisciplinary team convened to discuss the case and determine the course of treatment. The patient received TP chemotherapy in combination with trastuzumab monoclonal antibody, initiated 30-days post-surgery for four cycles. Subsequently, adjuvant radiotherapy consisting of 70 Gy was administered via intensity-modulated radiotherapy, concurrent with continued targeted therapy. Regular follow-ups were conducted to monitor the patient's progress (Fig. 1B‒H and Fig. 2B‒D). A total of 12 rounds of targeted therapy were administered, concluding in July 2022. The patient maintained stable disease throughout the treatment course. Based on their clinical characteristics and tumor biological behavior, we adopted a stratified follow-up strategy: comprehensive evaluations were conducted every 3-months within the first 2-years postoperatively, followed by follow-ups every 6-months from year 2 to year 5, and annual follow-ups thereafter. To date, no signs of tumor recurrence or metastasis have been identified after 36-months of standardized follow-up.

## Discussion

Compared with its parotid counterpart, SDC originating from the submandibular gland is relatively rare. Moreover, the management of de novo SDC ‒ arising without pre-existing pleomorphic adenoma ‒ has been reported only in isolated cases and small series.^2^ Currently, no standardized treatment protocol exists for tumors at this anatomical site, particularly regarding their response to multimodal therapy, including anti-HER2 targeted therapy combined with surgery or radiotherapy. In addition, most studies determine HER2 positivity solely through immunohistochemistry, lacking genetic analyses of amplification and mutations, which may limit the precision of therapeutic strategies. In this case, we systematically assessed both HER2 protein expression and gene status in an aggressive de novo SDC and documented the tumor’s dynamic changes during treatment, providing practical insights for individualized therapeutic approaches in similar clinical scenarios.

HER2 positivity constitutes a risk factor for breast cancer, correlating with increased aggressiveness and recurrence rates. Studies have demonstrated that trastuzumab extends overall survival in patients with HER2-positive breast cancer. Given the histological resemblance between salivary gland ductal carcinoma and breast cancer, HER2-targeting drugs such as trastuzumab are frequently employed in treating HER2-positive SDC. Trastuzumab has been reported to prolong the survival of patients with HER2-positive SDC. In a retrospective analysis by Kawakita et al., the median Overall Survival (OS) of HER2/AR-positive SDC patients receiving anti-HER2 treatment and androgen deprivation therapy was significantly longer than that of patients undergoing conventional treatment.^3^ However, the limited number of cases necessitates further confirmation through studies with larger sample sizes. In the present case, a comprehensive treatment plan encompassing local radiotherapy, chemotherapy, and targeted therapy yielded superior efficacy and enhanced quality of life compared to single-mode treatments. After a long-term follow-up, the patient remained relapse-free and achieved favourable outcomes.

High HER2 expression typically indicates a poor prognosis,^3^ but corresponding targeted therapies offer alternative treatment options. This holds significant implications for treatment strategies and prognosis evaluation. For instance, Toshiaki et al. demonstrated that high HER2 expression correlates with poor prognosis in SDC.^4^ Additionally, in a comparative study by Stevens et al., HER2 gene amplification was absent in 6 low-grade salivary duct carcinomas, whereas 13 out of 15 high-grade cases exhibited HER2 amplification. This suggests a correlation between HER2 gene amplification and SDC malignancy.^5^ Given the high rates of local recurrence and distant metastasis, patients with HER2-positive SDC require more frequent follow-up and more aggressive treatment approaches.

## Conclusion

This case report details the successful treatment of a patient diagnosed with HER2-positive salivary duct carcinoma. The patient demonstrated favorable responses to a combination of postoperative chemotherapy, trastuzumab-targeted therapy, and radiotherapy. Due to the identification of HER2 gene amplification in the tumor, a regimen combining anti-HER2-targeted medication with chemotherapy was administered. This treatment protocol was well-tolerated and ameliorated the patient's symptoms and prognosis.

## ORCID ID

Zongxuan He: 0000-0002-0934-1800

Wei Shang: 0000-0002-9726-3688

## Ethics approval and consent to participate

Ethics approval for this case report was not required according to the guidelines stated by the Research Ethics Board of Qingdao University.

## Data availability statement

The authors declare that all data are available in repository.

## Declaration of competing interest

The authors declare no conflicts of interest.
